# Well-Being During the COVID-19 Pandemic: The Roles of Age, Race, and Gender

**DOI:** 10.1093/geroni/igab046.1970

**Published:** 2021-12-17

**Authors:** Nicky Newton, Jennifer Lodi-Smith

## Abstract

In the early months of COVID-19, behavioral modifications (i.e., social distancing) were the only means available to ameliorate contagion. These had widespread ramifications for well-being, although older adults showed relatively less disruption and high resilience than their younger counterparts (Carney et al., 2021). Early findings highlight the need for a life course perspective when examining reactions to COVID-19, based on social structure, personal agency, and individual differences such as age, gender, and personality (Settersten et al., 2020). The presentations in this symposium contribute to a developing body of research that delves deeper into individual lived experiences during COVID-19. Using data from the Health and Retirement Study, Ryan examines cohort and age differences in pandemic-related social contact, communication, loneliness, and well-being for women in the US, revealing that the impact of pandemic-attributed psychosocial experiences on well-being differed by age group. Newton et al. examine associations between perceptions of future time, COVID-19 disruption, and psychological well-being among older Canadian women, finding that COVID-19 disruption moderated the relationship between constrained time horizons and well-being. Birditt and colleagues assessed racial disparities in relationships between COVID-related stress, social isolation, and depression among adults aged 18-97 from the Survey of Consumers, and found ethnic/racial minorities reported greater pandemic-related stress and that stress and social isolation had detrimental effects on well-being. A discussion by Lodi-Smith will emphasize the necessity to include individual differences – age, race, gender, cohort, cultural context –when examining pandemic-related well-being in order to provide a more nuanced body of research.

